# Cellular wall of *Eucalyptus grandis *under influence of growth regulators - chemical-anatomical aspects

**DOI:** 10.1186/1753-6561-8-S4-P95

**Published:** 2014-10-01

**Authors:** Regina Pereira, Maria Monteiro, Heber Abreu

**Affiliations:** 1 Universidade Federal Rural do Rio de Janeiro, Brazil

## 

This research was caried out to understand the behavior of growth regulators (giberelic acid (GA_3_) e 6-benzilaminopurin (BAP) on lignification system of *Eucalyptus grandis *(FORMER Hill Maiden). After GA_3 _(alone) and BAP (combined) applications, analyses were carried out. These regulators were applied by a atomization system at different concentrations levels. Anatomical analysis, including Mäule and Wiesner test as well as chemical analysis such as insoluble (Klason) and dioxane lignin were carried out as well. Infrared spectroscopy, methoxyl determination (Seizel Method) and total protein (Kjedahl Method) were performed. Synergism was suggested for G_[1]_C_[1] _treatment, at following concentrations: 49,13 μM and 111 μM. These treatments decreasing around 20% of lignin. In spite of infrared spectra that showed G/S composition, lignin GS, HG were confirmed by methoxyl content, suggesting regulators action on lignin biosynthesis and lignin structural modification. Positive Mäule and Wiesner test were observed, while protein did not modified significatively.

